# Serial Interval of COVID-19 among Publicly Reported Confirmed Cases

**DOI:** 10.3201/eid2606.200357

**Published:** 2020-06

**Authors:** Zhanwei Du, Xiaoke Xu, Ye Wu, Lin Wang, Benjamin J. Cowling, Lauren Ancel Meyers

**Affiliations:** University of Texas at Austin, Austin, Texas, USA (Z. Du, L.A. Meyers);; Dalian Minzu University, Dalian, China (X. Xu);; Beijing Normal University Computational Communication Research Center, Zhuhai, China (Y. Wu);; Beijing Normal University School of Journalism and Communication, Beijing, China (Y. Wu);; Institut Pasteur, Paris, France (L. Wang); University of Hong Kong, Hong Kong, China (B.J. Cowling);; Santa Fe Institute, Santa Fe, New Mexico, USA (L.A. Meyers)

**Keywords:** Wuhan, coronavirus, epidemiology, serial interval, China, severe acute respiratory syndrome coronavirus 2, SARS-CoV-2, 2019 novel coronavirus disease, COVID-19, viruses, respiratory infections, zoonoses

## Abstract

We estimate the distribution of serial intervals for 468 confirmed cases of coronavirus disease reported in China as of February 8, 2020. The mean interval was 3.96 days (95% CI 3.53–4.39 days), SD 4.75 days (95% CI 4.46–5.07 days); 12.6% of case reports indicated presymptomatic transmission.

Key aspects of the transmission dynamics of coronavirus disease (COVID-19) remain unclear (*1*). The serial interval of COVID-19 is defined as the time duration between a primary case-patient (infector) having symptom onset and a secondary case-patient (infectee) having symptom onset (*2*). The distribution of COVID-19 serial intervals is a critical input for determining the basic reproduction number (R_0_) and the extent of interventions required to control an epidemic (*3*).

To obtain reliable estimates of the serial interval, we obtained data on 468 COVID-19 transmission events reported in mainland China outside of Hubei Province during January 21–February 8, 2020. Each report consists of a probable date of symptom onset for both the infector and infectee, as well as the probable locations of infection for both case-patients. The data include only confirmed cases compiled from online reports from 18 provincial centers for disease control and prevention (https://github.com/MeyersLabUTexas/COVID-19).

Fifty-nine of the 468 reports indicate that the infectee had symptoms earlier than the infector. Thus, presymptomatic transmission might be occurring. Given these negative-valued serial intervals, COVID-19 serial intervals seem to resemble a normal distribution more than the commonly assumed gamma or Weibull distributions (*4*,*5*), which are limited to positive values (Appendix). We estimate a mean serial interval for COVID-19 of 3.96 (95% CI 3.53–4.39) days, with an SD of 4.75 (95% CI 4.46–5.07) days, which is considerably lower than reported mean serial intervals of 8.4 days for severe acute respiratory syndrome (*5*) to 14.6 days (*6*) for Middle East respiratory syndrome. The mean serial interval is slightly but not significantly longer when the index case is imported (4.06 [95% CI 3.55–4.57] days) versus locally infected (3.66 [95% CI 2.84–4.47] days), but slightly shorter when the secondary transmission occurs within the household (4.03 [95% CI 3.12–4.94] days) versus outside the household (4.56 [95% CI 3.85–5.27] days) (Figure). Combining these findings with published estimates for the early exponential growth rate COVID-19 in Wuhan (*7*), we estimate an R_0_ of 1.32 (95% CI 1.16–1.48) (*5*), which is lower than published estimates that assume a mean serial interval exceeding 7 days (*7*,*8*).

**Figure:**
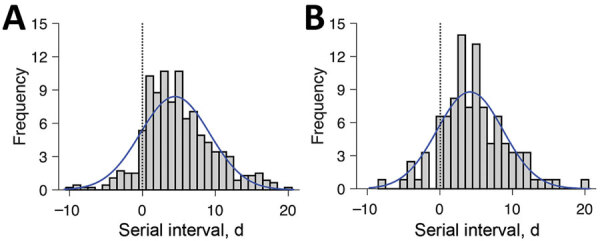
Estimated serial interval distribution for coronavirus disease (COVID-19) based on 468 reported transmission events, China, January 21–February 8, 2020. A) All infection events (N = 468) reported across 93 cities of mainland China as of February 8, 2020; B) the subset infection events (n = 122) in which both the infector and infectee were infected in the reporting city (i.e., the index patient’s case was not an importation from another city). Gray bars indicate the number of infection events with specified serial interval, and blue lines indicate fitted normal distributions. Negative serial intervals (left of the vertical dotted lines) suggest the possibility of COVID-19 transmission from asymptomatic or mildly symptomatic case-patients.

These estimates reflect reported symptom onset dates for 752 case-patients from 93 cities in China, who range in age from 1 to 90 years (mean 45.2 years, SD 17.21 years). Recent analyses of putative COVID-19 infector–infectee pairs from several countries have indicated average serial intervals of 4.0 days (95% CI 3.1–4.9 days; n = 28; unpub. data, H. Nishiura et al., unpub. data, https://doi.org/10.1101/2020.02.03.20019497), 4.4 days (95% CI 2.9–6.7 days, n = 21; S. Zhao et al., unpub. data, https://doi.org/10.1101/2020.02.21.20026559], and 7.5 days (95% CI 5.3–19, n = 6; *8*). Whereas none of these studies report negative serial intervals in which the infectee had symptoms before the infector, 12.6% of the serial intervals in our sample were negative.

We note 4 potential sources of bias. First, the data are restricted to online reports of confirmed cases and therefore might be biased toward more severe cases in areas with a high-functioning healthcare and public health infrastructure. The rapid isolation of such case-patients might have prevented longer serial intervals, potentially shifting our estimate downward compared with serial intervals that might be observed in an uncontrolled epidemic. Second, the distribution of serial intervals varies throughout an epidemic; the time between successive cases contracts around the epidemic peak (*9*). A susceptible person is likely to become infected more quickly if they are surrounded by 2 infected persons instead of 1. Because our estimates are based primarily on transmission events reported during the early stages of outbreaks, we do not explicitly account for such compression and interpret the estimates as basic serial intervals at the outset of an epidemic. However, if some of the reported infections occurred amid growing clusters of cases, then our estimates might reflect effective (compressed) serial intervals that would be expected during a period of epidemic growth. Third, the identity of each infector and the timing of symptom onset were presumably based on individual recollection of past events. If recall accuracy is impeded by time or trauma, case-patients might be more likely to attribute infection to recent encounters (short serial intervals) over past encounters (longer serial intervals). In contrast, the reported serial intervals might be biased upward by travel-related delays in transmission from primary case-patients that were infected in Wuhan or another city before returning home. If their infectious period started during travel, then we might be unlikely to observe early transmission events with shorter serial intervals. The mean serial interval is slightly higher for the 218 of 301 unique infectors reported to have imported cases.

Given the heterogeneity in type and reliability of these sources, we caution that our findings should be interpreted as working hypotheses regarding the infectiousness of COVID-19, requiring further validation. The potential implications for COVID-19 control are mixed. Although our lower estimates for R_0_ suggest easier containment, the large number of reported asymptomatic transmission events is concerning.

Appendix Additional information about serial interval of COVID-19 among publicly reported confirmed cases.
