# Supplementary material for: Serial Interval of COVID-19 among Publicly Reported Confirmed Cases
Source: Emerg Infect Dis. 2020 Jun;26(6):1341–3. doi: 10.3201/eid2606.200357 (PMC7258488; doi:10.3201/eid2606.200357)
Supplement: Appendix — Additional information about serial interval of COVID-19 among publicly reported confirmed cases. [file 20-0357-Techapp-s1.pdf]

Article DOI: https://doi.org/10.3201/eid2606.200357

Serial Interval of COVID-19 among Publicly Reported Confirmed Cases

Appendix

Data

We collected publicly available data on 6,903 confirmed cases from 271 cities of mainland China, that were available online as of February 8, 2020. The data were extracted in Chinese from the Web sites of provincial public health departments and translated to English (https://github.com/MeyersLabUTexas/COVID-19). We then filtered the data for clearly indicated transmission events consisting of: (i) a known *infector* and *infectee*, (ii) reported locations of infection for both cases, and (iii) reported dates and locations of symptom onset for both cases. We thereby obtained 468 infector–infectee pairs identified via contact tracing in 93 Chinese cities between January 21, 2020 and February 8, 2020 (Appendix Figure 1). The index cases (infectors) for each pair are reported as either importations from the city of Wuhan (N = 239), importations from cities other than Wuhan (N = 106) or local infections (N = 122). The cases included 752 unique individuals, with 98 index cases who infected multiple people and 17 individuals that appear as both infector and infectee. They range in age from 1 to 90 years and include 386 females, 363 males and 3 cases of unreported sex.

Inference Methods

Estimating Serial Interval Distribution

For each pair, we calculated the number of days between the reported symptom onset date for the infector and the reported symptom onset date for the infectee. Negative values indicate that the infectee developed symptoms before the infector. We then used the fitdist function in Matlab (*1*) to fit a normal distribution to all 468 observations. It finds unbiased estimates of the mean and standard deviation, with 95% confidence intervals. We applied the

same procedure to estimate the means and standard deviations with the data stratified by whether the index case was imported or infected locally.

Estimating the Basic Reproduction Number (***R***$_0$)

Given an epidemic growth rate *r* and *normally distributed generation times* with mean (μ) and standard deviation (σ), the basic reproduction number is given by

$R_0 = e^{r\mu-(1/2)r^2\sigma^2}$ (*2*).

Since we do not know the COVID-19 generation time distribution, we use our estimates of the COVID-19 serial interval distribution as an approximation (Appendix Table 1), noting that the serial interval distribution tends to be more variable than the generation time distribution. We assume that the COVID-19 growth rate (*r*) is 0.10 per day [95% CI 0.050–0.16] based on a recent analysis of COVID-19 incidence in Wuhan, China (*3*). To estimate R_0, we take 100,000 Monte Carlo samples of the growth rate ($r \sim N(.1, 0.028)$) and the mean and standard deviation of the serial interval ($(\mu, \sigma) \sim N(\mathbf{M}, \Sigma)$ where $\mathbf{M} = (3.96, 4.75)$ and $\Sigma = \begin{bmatrix} 0.048 & 2.4{\cdot}10^{-18} \\ 2.4{\cdot}10^{-18} & 0.24 \end{bmatrix}$). We thereby estimate an R_0 of 1.32 [95% CI 1.16–1.48].

Model Comparison

We used maximum likelihood fitting and the Akaike information criterion (AIC) to evaluate four candidate models for the COVID-19 serial interval distributions: normal, lognormal, Weibull and gamma. Since our serial interval data includes a substantial number of non-positive values, we fit the four distributions both to truncated data in which all non-positive values are removed and to shifted data in which 12 days are added to each observation (Appendix Figure 1, Appendix Tables 2, 3). The lognormal distribution provides the best fit for the truncated data (followed closely by the gamma and Weibull). However, we do not believe there is cause for excluding the non-positive data and would caution against making assessments and projections based on the truncated data. The normal distribution provides the best fit for the full dataset (shifted or not) and thus is the distribution we recommend for future epidemiologic assessments and planning.

Supplementary Analysis

To facilitate interpretation and future analyses, we summarize key characteristics of the COVID-2019 infection report dataset.

Age Distribution

Of the 737 unique cases in the dataset, 1.7%, 3.5%, 54.1%, 26.1% and 14.5% were ages 0–4, 5–17, 18–49, 50–64, and over 65 years, respectively. Across all transmission events, approximately one third occurred between adults ages 18 to 49, ~92% had an adult infector (over 18), and over 99% had an adult infectee (over 18) (Appendix Table 4).

Secondary Case Distribution

Across the 468 transmission events, there were 301 unique infectors. The mean number of transmission events per infector is 1.55 (Appendix Figure 2) with a maximum of 16 secondary infections reported from a 40 year old male in Liaocheng city of Shandong Province.

Geographic Distribution

The 468 transmission events were reported from 93 Chinese cities in 17 Chinese provinces and Tianjin (Appendix Figure 3). There are 22 cities with at least five infection events and 71 cities with fewer than five infection events in the sample. The maximum number of reports from a city is 72 for Shenzhen, which reported 339 cumulative cases as of February 8, 2020.

References

19. MathWorks. Fit probability distribution object to data—MATLAB fitdist [cited 2020 Feb 19]. https://www.mathworks.com/help/stats/fitdist.html

20. Wallinga J, Lipsitch M. How generation intervals shape the relationship between growth rates and reproductive numbers. Proc Biol Sci. 2007;274:599–604. PubMed https://doi.org/10.1098/rspb.2006.3754

21. Li Q, Guan X, Wu P, Wang X, Zhou L, Tong Y, et al. Early transmission dynamics in Wuhan, China, of novel coronavirus-infected pneumonia. N Engl J Med. 2020;NEJMoa2001316; Epub ahead of print. PubMed https://doi.org/10.1056/NEJMoa2001316

Appendix Table 1. Model comparison for COVID-19 serial intervals based on 122 reported transmission events in China between January 21, 2020 and February 8, 2020 in which both the infector and infectee were infected in the reporting city (i.e., the index case was not an importation from another city)

Data	Distribution	Mean/Shape (95% CI)	SD/Scale (95% CI)	AIC
Original data	Normal (Mean, SD)	3.66 (2.84– 4.47)	4.54 (4.03–5.20)	718.45
Truncated (>0)	Normal (Mean, SD)	5.21 (4.47–5.95)	3.66 (3.20–4.26)	524.34
	Lognormal (Shape, Scale)	2.14 (1.64–2.78)	2.44 (1.81–3.28)	486.81
	Gamma (Shape, Scale)	1.40 (1.25–1.55)	0.75 (0.65–0.87)	487.68
	Weibull (Shape, Scale)	5.81 (5.05–6.68)	1.52 (1.30–1.77)	489.59
Shifted (+12d)	Normal (Mean, SD)	13.66 (12.84– 14.47)	4.54 (4.03– 5.20)	718.45
	Lognormal (Shape, Scale)	7.52 (5.88–9.61)	1.82 (1.41–2.34)	730.75
	Gamma(Shape, Scale)	2.55 (2.47–2.62)	0.41 (0.37–0.47)	755.39
	Weibull(Shape, Scale)	15.19 (14.32–16.11)	3.18 (2.79–3.63)	722.22

Appendix Table 2. Estimated serial interval distributions based on the location of index infection (imported versus local) and the secondary infection (household versus nonhousehold)*

Group	Mean (95 CI%)	SD (95 CI%)	Proportion of serial intervals <0
All (N = 468)	3.96 (3.53–4.39)	4.75 (4.46–5.07)	12.61% (N = 59)
Locally infected index case (n = 122)	3.66 (2.84–4.47)	4.54 (4.03–5.20)	14.75% (N = 18)
Imported index case (n = 346)	4.06 (3.55–4.57)	4.82 (4.48–5.21)	11.85% (N = 41)
Household secondary infection (n = 104)	4.03 (3.12–4.94)	4.69 (4.12–5.43)	16.35% (N = 17)
Nonhousehold secondary infection (n = 180)	4.56 (3.85–5.27)	4.80 (4.35–5.36)	11.11% (N = 20)

*We assume that the serial intervals follow normal distributions and report the estimated means and standard deviations for all 468 infector–infectee pairs reported from 93 cities in mainland China by February 8, 2020, 122 pairs in which the index case was infected locally, 346 pairs in which the index case was an importation from another city, 104 pairs in which the secondary transmission event occurred within a household, and 180 pairs in which the secondary transmission event was reported as non-household. The rightmost column provides the proportion of infection events in which the secondary case developed symptoms before the index case.

Appendix Table 3. Model comparison for COVID-19 serial intervals based on all 468 reported transmission events in China between January 21, 2020 and February 8, 2020.

Data	Distribution	Mean/Shape (95% CI)	SD/Scale (95% CI)	AIC
Original data	Normal (Mean, SD)	3.96 (3.53– 4.39)	4.75 (4.46– 5.07)	2,788.77
Truncated (>0)	Normal (Mean, SD)	5.62 (5.21–6.03)	3.92 (3.66–4.23)	2,014.12
	Lognormal (Shape, Scale)	2.02 (1.76–2.31)	2.78 (2.39–3.25)	1,886.73
	Gamma (Shape, Scale)	1.46 (1.38–1.54)	0.78 (0.72–0.84)	1,898.63
	Weibull (Shape, Scale)	6.25 (5.81–6.72)	1.50 (1.38–1.62)	1,892.04
Shifted (+12d)	Normal (Mean, SD)	15.96 (15.53–16.39)	4.75 (4.46–5.07)	2,788.77
	Lognormal (Shape, Scale)	9.83 (8.66–11.15)	1.62 (1.43–1.85)	2,822.71
	Gamma(Shape, Scale)	2.72 (2.69–2.75)	0.35 (0.33–0.38)	2,898.24
	Weibull(Shape, Scale)	17.66 (17.19–18.14)	3.56 (3.32–3.80)	2,806.21

Appendix Table 4. Age distribution for the 457 of 468 infector–infectee pairs*

	Infectee age group, y					
Infector age group, y	0–4	5–17	18–49	50–46	>65	Total
0–4	0	0	0	0	0	0
5–17	0	0	1	0	1	2
18–49	12	18	154	60	44	288
50–46	1	5	47	49	13	115
>65	0	1	22	10	19	52
Total	13	24	224	119	77	457

*Each value denotes the number of infector–infectee pairs in the specified age combination. Age was not reported for the remaining 11 pairs.

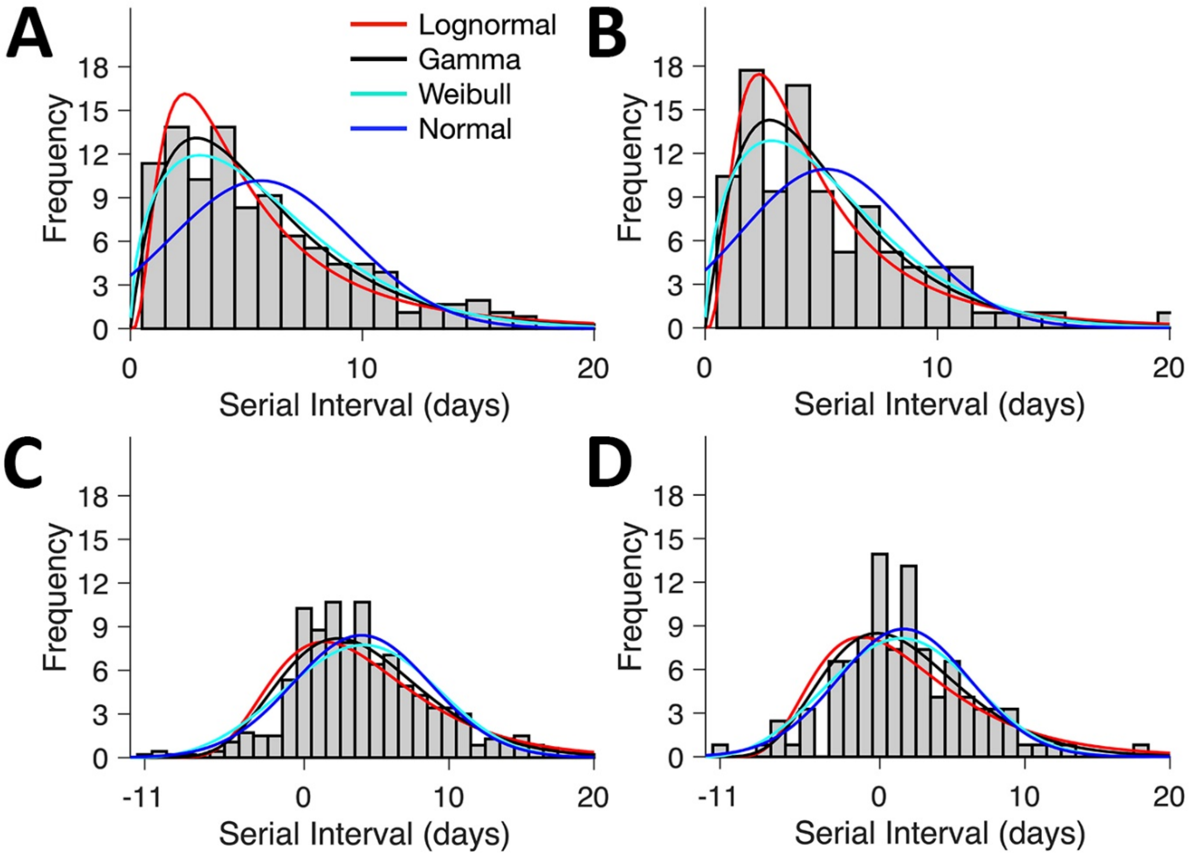

Appendix Figure 1. Maximum likelihood distributions fit to transformed COVID-19 serial intervals (468 reported transmission events across 93 cities in Mainland China between January 21, 2020 and February 8, 2020). To evaluate several positive-valued distributions (lognormal, gamma and Weibull), we took two approaches to addressing the negative-valued data. First, we left truncated the data (i.e., removed all non-positive values) for (a) all 468 infection events and (b) the subset of infection events (N = 122) in which both the infector and infectee were infected in the reporting city (i.e., the index case was not an importation from another city). Second, we shifted the data by adding 12 days to each reported serial interval for (c) all infection events and (d) the subset of infection events in which both the infector and infectee were locally infected. Bars indicate the number of infection events with the specified serial interval and colored lines indicate the fitted distributions. Parameter estimates and AIC values are provided in Appendix Table 3.

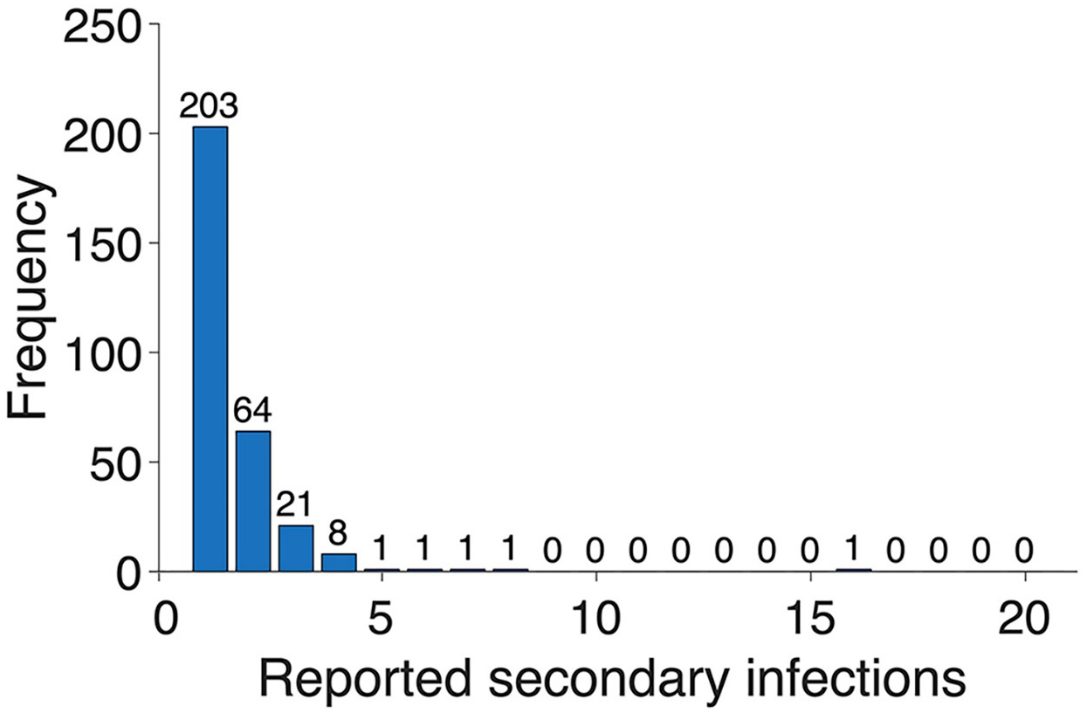

Appendix Figure 2. Number of infections per unique index case in the infection report dataset. There are 301 unique infectors across the 468 infector-infectee pairs. The number of transmission events reported per infector ranges from 1 to 16, with ~55% having only one.

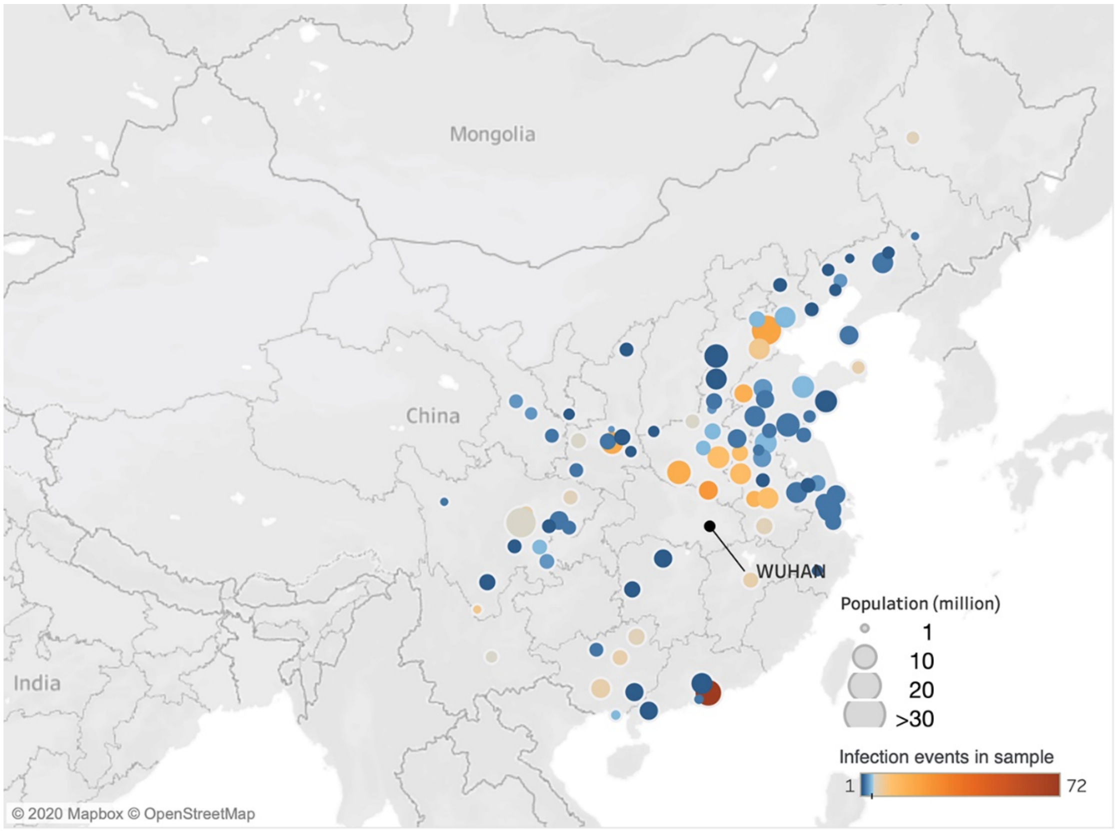

Appendix Figure 3. Geographic composition of the infection report dataset. The data consist of 468 infector-infectee pairs reported by February 8, 2020 across 93 cities in mainland China. Colors represent the number of reported events per city, which range from 1 to 72, with an average of 5.03 (SD 8.54) infection events. The 71 cities with fewer than five events are colored in blues; the 22 cities with at least five events are colored in shades of orange.
